# Measuring and Predicting Maturity to Parenthood: What Has Personality Got to Do with It?

**DOI:** 10.3390/jcm10245802

**Published:** 2021-12-11

**Authors:** Ariadna Beata Łada-Maśko, Maria Kaźmierczak

**Affiliations:** 1 Division of Developmental Psychology and Psychopathology, Institute of Psychology, Faculty of Social Sciences, University of Gdańsk, 80-309 Gdańsk, Poland; 2 Division of Family Studies and Quality of Life, Institute of Psychology, Faculty of Social Sciences, University of Gdańsk, 80-309 Gdańsk, Poland; maria.kazmierczak@ug.edu.pl

**Keywords:** maturity, parenthood, empathy, resiliency, life values, young adults, individual differences, close relationship

## Abstract

Maturity to parenthood is essential for taking on parental roles but remains an understudied issue. Still, close relations between maturity and personality dimensions are commonly emphasized. Thus, conducting research on maturity in context of personality seems a valuable research direction. The present research consists of two studies, focusing on the development and validation of Maturity to Parenthood Scale (MPS), in relation to personality, emotional regulation, coping with challenges, and intimate relationship satisfaction. In both studies, childless adults aged 20–35 years took part: (1) 718 participants (*M*_age_ = 25.49; *SD* = 2.89; 479 women), (2) 150 participants (*M*_age_ = 23.69; *SD* = 3.15; 104 women). All the participants had been in an intimate relationship for at least six months at the time of the study, the majority declared their willingness to have children in the future, had higher education, and were professionally active. The results showed that MPS is a reliable, valid measure comprising the following three subscales: valence, behavioral, and cognitive–emotional maturity to parenthood. The findings also confirmed the importance of broad- and narrow-band individual differences and contextual factors for maturity. MPS may be used in psychoeducation, supporting the transition to biological or adoptive/foster parenthood, as well as in medical and psychological care.

## 1. Introduction

Maturity is widely explored as a concept, but most studies focus on the general individual maturity of a person, and maturity for marriage or to carry out parental roles [1,2,3,4,5]. Maturity for marriage and parenthood is defined as an appropriate level of intellectual, personality, emotional, and social development, which are intertwined [1,6,7,8,9,10,11,12]. Moreover, gender differences in maturity referring to parenthood have also been found, confirming higher maturity in women [1,8,13,14,15].

Before deciding to become a parent, young adults estimate the investments related to having a child and the limitations resulting from the new role [16,17,18]. There has been a shift in women of the highest fertility from the 20–24-year age group to the 25–29-year age group. Furthermore, fertility has significantly increased in the age group of 30–34 years, which is mainly due to the implementation of postponed parenthood [19,20]. Thus, observing current sociocultural changes and trends in marriage and fertility [19,21,22,23], it seems extremely important to explore maturity to parenthood in young adults. Studies have revealed that psychoeducation and clarity in visualizing themselves as future parents majorly impact adjustment to pregnancy and fulfilling future parental roles [24,25,26]. Moreover, the quality of a close relationship, good communication, and mutual support help couples to consciously prepare for parenthood, reducing the level of stress experienced related to the transition to parenthood as a new stage in family life [6,27,28,29]. The positive effects of psychoeducation and the relationship quality on the mental health of expecting couples have also been confirmed [30,31,32]. Therefore, conducting research on maturity to parenthood and thus identifying areas where young adults need further support is a valuable research direction in the context of the role of personality for clinical application.

Surprisingly, maturity to parenthood appears in the literature primarily in the context of couples who have already had children [21,33,34,35]. Thus, existing definitions mainly focus on the fulfillment of parental roles [36,37,38,39]. Tate and Patterson [40] recently used a term closely related to maturity to parenthood, “parenthood aspirations”, which involves the following three aspects of parenthood: desires, expectations, and intentions. The parenthood desires aspect refers to how much people want to become a parent, the parenthood expectations aspect considers the likelihood of thinking about becoming parents, and the parenthood intentions aspect is about planning to pursue parenthood. In this paper, we focus on the perspective of personality psychology; we refer to the definition proposed by Bakiera, who defined parental involvement as referring to psychological readiness. First, this concept distinguishes commitment in the area of behavior; second, the importance of the role of a parent in a person’s values system, then to cognitive and affective aspects, and, accordingly, concentrating thoughts, attention, and imagination on parenting roles and emotional concentration on parenthood.

On the basis of the above conceptual framework [36,40], maturity to parenthood could be considered to be the readiness for taking on future parenting roles in the following three dimensions: valence, behavioral, and cognitive–emotional maturity to parenthood. The first dimension, valence maturity to parenthood, refers to placing parenthood in a person’s value system, the motives of their parental aspirations, and creating a vision of parenthood based on their ethical and moral norms. The second dimension of behavioral maturity to parenthood concerns a person’s activities in intimate relationships and in other social relationships to take on the future parental roles, their activities in the economy and work, and actively seeking information about parenthood. The last dimension, cognitive–emotional maturity to parenthood, refers to a general approach for taking and implementing parental roles in everyday life, drawing attention to the different aspects of parenthood, creating a picture of parental roles in the context of a person’s family of origin, emotions related to parenthood, and the responsibility for the decisions affecting the implementation of parental roles in the future. 

When exploring maturity to parenthood, both intra- and interindividual factors should be considered. The role of broad-band (Big Five) and narrow-band (e.g., empathy) personality dimensions in a wide range of parental behaviors, including the parent–child relationship, are commonly emphasized [8,25,36,41,42,43]. Research has shown that high neuroticism in mothers correlates with negative reactions to their child, stress, and depression [44]. Extraversion is associated with greater involvement in parent–child contact, displaying positive affection and encouraging children to explore the world [45,46]. Agreeableness is a close parent–child relationship factor based on support and cooperation [8,43]. Furthermore, higher levels of agreeableness predict lower levels of negative affect and intrusive–overcontrolling behavior toward children [45,46,47]. Studies have also revealed that openness to experiences is related to positive parenting for both mothers and fathers, as being more open-minded, imaginative, and reflective could favor good parenting [45,47]. For instance, parents who are high in the openness dimension are more likely to provide their children with a stimulating environment, and to be more flexible and open to modern parenting approaches [48]. However, parents too high in the openness to experience dimension may also be more focused on their own interests and aspirations instead of being engaged and responsive parents [47]. Conscientiousness is also positively related to supportive parenting, and negatively to controlling parenting [49]. Moreover, studies have shown that more conscientious parents provide a more structured and consistent environment for their children [48] and show more positive emotions and greater responsiveness toward children [47,50]. 

Subsequently, as this article focuses on the maturity to take on the socially highly regarded but stressful role of a parent [51,52,53], narrow-band personality dimensions that are related to emotions and cognition (e.g., individual differences in empathy) and coping with difficulties as challenges (e.g., resiliency) exert significant effects on the adaptation to parental roles [8,10,25]. Moreover, directed at others, emotional and cognitive empathy (referred to in the literature as empathic concern and perspective taking) is strongly associated with emotional maturity [54], as it involves care and consideration of the points of view of others in various social situations [55]. Empathy, thus, facilitates the expression of positive emotions and dealing with negative emotions, intimacy in close relationships, and mutual support and understanding needs or problems in relationships, thereby coping with various social challenges [54,56]. However, taking on and experiencing others’ negative emotions (referred to as personal distress in the literature) has been linked to a lack of emotional regulation and difficulties in relationships [56].

The transition to parenthood induces changes in many areas of life and is thus commonly associated with a number of stressors [57,58]. The most common sources of parental stress are a child’s difficult temperament, the relationship between parents and their child, perceptions of parental roles, differences between imagined parenthood and reality, and the life situation after the baby is born [59,60]. Furthermore, parental stress is connected to lower parental sensitivity [61]. However, research shows that resiliency can facilitate coping with parental responsibilities and can improve parents’ wellbeing, both emotional and physical, and thus the wellbeing of their children, albeit indirectly [62].

Lastly, contextual variables are important in parenting and are worth exploring in the analysis of maturity to parenthood. The perception of parenting and taking on maternal and paternal roles are largely based on the quality of an intimate relationship with a partner [36]. Studies have also revealed that the quality of a close relationship is an important protective factor in coping with parenting stress [59,63]. 

### The Present Research

Maturity to parenthood is a key issue when starting a family to ensure good preparation for taking on parenting roles and coping with the challenges of raising a child. However, little is known about maturity to parenthood referring to a group of childless adults, thus ignoring an extremely important aspect of the readiness to take on parental roles in the future. Moreover, previous considerations regarding maturity to parenthood have not taken into account the multidimensionality of the construct. Within the above theoretical and practical framework, the present research focuses on the development and validation of the Maturity to Parenthood Scale (MPS), which may help to assess maturity to parenthood and highlight areas where young adults need further support. The above measure may also be helpful in assessing candidates to be adoptive or foster parents. It may also be used in psychoeducation to prevent problematic parental behaviors in future parents.

The present investigation involved two studies on the development and validation of the MPS. In Study one, we focused on defining the construct of maturity to parenthood with factor and reliability analysis of the MPS. Study two was designed to provide initial evidence regarding the stability and validity of the MPS, involving correlates of the MPS. Therefore, this research presents the results on the psychometric properties of the MPS, its reliability and validity in relation to personality, emotional regulation, coping with challenges, and intimate relationship satisfaction.

## 2. Procedure

This project was approved by the Ethics Board for Research Projects at the Institute of Psychology, University of Gdansk, Poland (decision no. 7/2018). Participants were recruited via announcements on social media or in various interest groups for young adults. The inclusion criteria for the study were age (in the range of 20–35 years, i.e., young adulthood), being in a current relationship for at least six months, and not having had children yet. Consent to participate in the study was collected from all the participants, and they were informed that participation in this study was voluntary and that they could withdraw at any time. They were also informed that the results of this study would only be used for scientific purposes. The set of questionnaires was provided via an online platform to which the participants received a link. No sensitive personal data were gathered. No payment was provided for participation in the study. The procedure was the same for Studies one and two.

## 3. Study 1

Considering the above-mentioned theoretical framework [36,40], the aim of Study one was to define the construct of maturity to parenthood and to investigate the structure and reliability of the newly proposed measure, the Maturity to Parenthood Scale (MPS). The following hypotheses were formulated:

**Hypothesis** **1** **(H1).** *The MPS has a three-dimensional structure: valence, behavioral, and cognitive–emotional maturity to parenthood.*

**Hypothesis** **2** **(H2).** *Overall maturity to parenthood and its subscales are characterized by high reliability.*

Additionally, we explored whether women were characterized by a higher overall level of maturity to parenthood and a higher level of its three aspects: valence, behavioral, and cognitive–emotional maturity to parenthood.

### 3.1. Participants

In total, 718 young childless adults aged 20–35 years (*M*_age_ = 25.49; *SD* = 2.89; 479 women) participated in the study. The average age of the women was 23 years old (*M*_age_ = 23.12; *SD* = 2.65), whereas the average age of the men was 24 years old (*M*_age_ = 24.25; *SD* = 2.65). All the study participants had been in their current relationship for at least six months (*M* = 3.5; *SD* = 2.38; years), and 7.8% were married; 50.2% of the young adults lived with a partner. Importantly, from the perspective of the present study, 90.8% of the young adults declared their willingness to have children in the future. The majority of the research group had higher education (73.8%) and worked (67.7%). Furthermore, 21.6% of the study participants had secondary education, 4.5% had vocational education, and only 0.1% of participants had only primary education.

### 3.2. Defining the Constructs and Generating Test Items

The first stage in the development of the MPS was defining maturity to parenthood. On the basis of analyzing the scientific literature, previous research on parenting (see Section 1), and primarily on the basis of Bakiera’s [36] concept of involved parenting, definitions of maturity to parenthood in the three areas, namely, valence, behavior, and cognitive–emotional maturity to parenthood, were prepared (see descriptions in Section 4.3.1). Then, an exploratory study was carried out. Ten competent judges (psychologists from various age groups with practical and scientific experience in the areas of developmental, family, or clinical psychology) were presented with definitions of the components of maturity to parenthood and were asked to provide examples of statements describing them. On the basis of the analysis of the obtained information and content verification of similar statements, the original version of the 105 statements was developed. Subsequently, the statements were presented to another set of 10 competent judges (as well as previously—specialists with practical and scientific experience in the areas of developmental, family, or clinical psychology), who were asked to assess the adequacy of each item to the three definitions of the components of maturity to parenthood on a seven-point scale from –3 (“does not fit the definition at all”) through 0 (“neither meets nor does not meet the definition”), to +3 (“it fully meets the definition”). On the basis of the judges’ assessment of the content validity ratio (CVR) [64,65], 45 items for the three components were selected as the most congruent with the following definitions: Valence maturity to parenthood with 12 items, behavioral maturity to parenthood with 14 items, and cognitive–emotional maturity to parenthood with 19 items. The level of inter-judge agreement was medium (W-Kendall = 0.57). 

### 3.3. Statistical Analysis

Statistical analysis was conducted using a Statistical Package for the Social Sciences (SPSS) version 26 (SPSS Inc. (IBM Corp, Armonk, NY, USA), license purchased by the University of Gdansk and MPLUS 7.2 software. Confirmatory factor analysis (CFA) in MPLUS 7.2 using the WLSMV estimator (weighted least squares with adjusted means and variances) was conducted to verify the original factorial structure of the MPS according to the theoretical background. CFA was carried out according to the following recommendations for analyzing fit indices [66,67]: Comparative fit index (CFI) of 0.90–0.95 = acceptable model fit and of >0.95 = good model fit; Tucker–Lewis index (TLI) of 0.90–0.95 = acceptable model fit and of >0.95 = good model fit; root mean square error of approximation (RMSEA) of ≤0.05 = good fit, of <0.08 = acceptable fit, and of ≥0.10 = poor fit.

### 3.4. Results

#### 3.4.1. Structure of the MPS

CFA confirmed the three-factor structure of the MPS; we also attempted one- and four-factor structures (cognitive–emotional factor divided into the two factors of cognitive and emotional separately), but the three-factor structure of the MPS was the best fit (see Table 1). The results also indicated the necessity to revise scales and remove some items. Items with an estimation power under 0.5 were excluded. The standardized item loadings are shown in Table 2. The model fit indices of the presented model and the overall fit of the model were satisfactory: χ^2^ = 19739.24, df = 276, *p* < 0.001; CFI = 0.94, TLI = 0.94, and RMSEA (90% CI) = 0.079 (0.075–0.083); thus, the final model of the MPS consisted of 24 items.

Furthermore, all the MPS subscales were correlated (see Table 3).

#### 3.4.2. The Reliability of the MPS

The reliability of the MPS was examined by separately calculating the alpha coefficient for each subscale and for the overall score. Coefficient values above 0.70 are recommended for instruments used in the health and social sciences or for research purposes [68,69], and all Cronbach’s alpha coefficients were above 0.85 for the MPS (see Table 4). 

#### 3.4.3. Sex and Maturity to Parenthood of Young Adults

The differences between women and men on maturity to parenthood were assessed using Student’s *t*-test for independent samples. The results are presented in Table 5.

### 3.5. Discussion

The results of Study one confirmed the three-dimensional structure and reliability of the Maturity to Parenthood Scale. However, a few modifications were made to improve the proposed measure. First, the uni- and four-dimensional models with 45 items were tested. The unidimensional model had a poor fit to the data, whereas the four-dimensional model had an acceptable fit. However, we assumed that the model should be supported with the theoretical background, and we also tried the three-dimensional model, which had the best fit to the data. This model was based on the structure proposed by Bakiera [36], who distinguished the valence, behavioral, and cognitive–emotional aspects of parental involvement (see Section 1). We also removed 21 items from the primary list with estimation power under 0.5 to provide the most valuable measure. Lastly, the MPS consisted of 24 items. The correlations between the three dimensions of maturity to parenthood were strong (0.66 and higher), which confirmed that they measured the same construct in different aspects [36]. Furthermore, our reliability results suggest that the MPS can be a very useful instrument for assessing maturity to parenthood in young adults. Additionally, the results showed that women presented a higher level of maturity to parenthood than men in all three dimensions, as well as in the overall score of the maturity to parenthood. However, despite the applied statistical procedures and the reliability of the obtained results, it should also be noted that significantly more women than men took part in the study, which could have had a possible impact on the results. Still, the obtained results confirmed earlier findings regarding differences between women and men in the parenting domain. Thus, the above results are consistent with those of Todosijević and Ignjatović [15], who reported that women are expected to be more mature to parenthood and to reach maturity earlier than men. The differences in maturity to parenthood between men and women can be also explained through sociocultural factors. The research conducted by Kaźmierczak and Karasiewicz [14] confirmed that Polish men identify with the role of father less than women do with the role of mother. Furthermore, the paternal role seems to be shaped by both individual and interpersonal factors (relationships with a partner), whereas for women, in shaping their parental role, relationships with a partner are less important [25,70]; thus, when analyzing men’s maturity to parenthood, it is valuable to also analyze the quality of their close relationships. 

## 4. Study 2

The aim of Study two was to better describe the construct of maturity to parenthood; therefore, the stability of the MPS as well as its correlates were examined.

On the basis of the above literature review, we expected that both the general personality dimensions and the contextual variables (related to functioning in close relationships and the perception of taking future parental roles) would be related to overall maturity to parenthood and its dimensions. Moreover, we predicted that, due to the complex nature of the maturity to parenthood construct, the MPS dimensions would also be related to a person’s life values, narrow-band personality dimensions (e.g., cognitive and emotional empathy), and resistance to difficult situations and coping with challenges, one of which might be parenthood. 

Thus, the following hypotheses were formulated:

**Hypothesis** **3** **(H3).** *The Maturity to Parenthood Scale has satisfactory stability.*

**Hypothesis** **4** **(H4).** *The overall level and three dimensions of maturity to parenthood are associated with general personality dimensions and the quality of current close relationships, as well as declared willingness to have children in the future.*

**Hypothesis** **5** **(H5).** *Valence maturity to parenthood is especially related to an individual’s life values.*

**Hypothesis** **6** **(H6).** *Behavioral maturity to parenthood is especially associated with resiliency.*

**Hypothesis** **7** **(H7).** *Cognitive–emotional maturity to parenthood is especially associated with the emotional and cognitive dimensions of empathy.*

Additionally, we expected other associations between the overall score and the three dimensions of maturity to parenthood, as well as the studied variables.

### 4.1. Participants

The stability and validity of the MPS were tested in Study two. In total, 150 young childless adults aged 20–35 years (*M*_age_ = 23.69; *SD* = 3.15; 104 women) participated in the study. All the study participants had been in their current relationship for at least six months (*M* = 3.66 years; *SD* = 2.44 years), and 9.3% were married. Furthermore, 49.3% of the young adults lived with their partner, and 92% declared their willingness to have children in the future. The majority of the research group had higher education (89.3%) and worked (68%).

### 4.2. The Stability of the MPS

The stability of the MPS was tested using the test–retest method. The participants were tested twice with the MPS, three months apart. The correlations between the first and second measurements were as follows: valence maturity to parenthood, *r* = 0.88; behavioral maturity to parenthood, *r* = 0.85; cognitive–emotional maturity to parenthood, *r* = 0.87; and overall maturity to parenthood, *r* = 0.90. Thus, the MPS has satisfactory stability.

### 4.3. The Validity of the MPS

#### 4.3.1. Measures

##### Maturity to Parenthood Scale 

The MPS *(Łada-Maśko, Kaźmierczak; Appendix A)* self-report questionnaire consists of 24 items with a seven-point Likert response scale (1 = strongly disagree; 7 = strongly agree). The overall score is the sum of the obtained points from all the statements. The above scale consists of the following three subscales, for which the results for maturity to parenthood in particular areas can also be calculated:

1. Valence maturity to parenthood (VMP; seven items): High scores in this subscale are obtained by a person who places parenting high in their coherent and integrated value system, has a correct and critical insight into the motives of their parental aspirations, and is also based on their ethical and moral norms, creating a vision of their own parenthood.
2. Behavioral maturity to parenthood (BMP; nine items): High scores on this subscale mean that a person directs their activities in intimate relationships and in other social relationships to take on future parental roles, directs their activities in the economic and work spheres to take on future parental roles, and actively seeks information about parenthood.
3. Cognitive–emotional maturity to parenthood (CEMP; eight items): The person who scores high in this subscale presents a realistic and flexible approach for taking on and implementing parental roles in everyday life, draws attention to the different aspects of parenthood, creates a picture of parental roles in the context of their family of origin, and presents positive emotions regarding the vision of being a parent. Furthermore, this person feels responsible for the choices and decisions they make, which may affect the future implementation of parental roles.

Cronbach’s α values for the subscales in this study were as follows: valence maturity to parenthood = 0.85, behavioral maturity to parenthood = 0.88, and cognitive–emotional maturity to parenthood = 0.87. Furthermore, reliability for overall maturity to parenthood was α = 0.94.

##### Portrait Values Questionnaire (PVQ-RR)

The Polish adaptation of the Portrait Values Questionnaire (PVQ-RR) by Cieciuch [71,72] was used to measure life values. The questionnaire consists of 57 items that are evaluated on a six-item Likert-type scale, where the respondents assess if the described person is 1 = quite unlike me; 2 = unlike me; 3 = a bit similar to me; 4 = on average, similar to me; 5 = similar to me; 6 = very similar to me. The 19 narrowly defined values in the questionnaire are self-direction thought, self-direction action, stimulation, hedonism, achievement, power dominance, power resources, face, security personal, security societal, tradition, conformity—rules, conformity—interpersonal, humility, universalism—nature, universalism—concern, universalism—tolerance, benevolence—care, and benevolence—dependability. The reliability for this questionnaire in this study was α = 0.94.

##### Assessment of Resiliency Scale

The Assessment of Resiliency Scale (SPP-25) [58,73] consists of 25 items. The scale allows for measuring the overall level of resiliency and its five constituent factors, namely, perseverance and proactive approach, personal coping skills and tolerance of negative emotions, openness to new experiences and sense of humor, optimistic attitude to life, and the ability to mobilize oneself in difficult situations, tolerance of failures, and treating life as a challenge. The items are evaluated on a five-item Likert-type scale from 0 (strongly disagree) to 4 (strongly agree). The reliability for this scale in the present study was α = 0.90.

##### Empathic Sensitiveness Scale (SWE) 

The SWE [74] is a 28-item scale to measure dispositional empathy. The scale contains three subscales that measure the following three components of empathy: empathic concern, personal distress, and perspective taking. The items are evaluated on a five-item Likert-type scale from 1 (strongly disagree) to 5 (strongly agree); respondents declare if they agree with a statement or not. The Cronbach’s α in the present study was 0.80 for empathic concern, 0.78 for personal distress, and 0.77 for perspective taking.

##### Relationship Evaluation Questionnaire

To measure relationship satisfaction, the Polish adaptation of the Relationship Evaluation Questionnaireby Rostowska and Kaźmierczak ([75]; RELAT, unpublished scale) was used. This questionnaire is a seven-item measure of relationship satisfaction, with a five-point Likert-type response scale. With this tool, we asked participants how satisfied they were with various aspects of their relationship (i.e., physical intimacy, time together, their ways of solving problems, and relationship equality). The Cronbach’s α for RELAT in the present study was 0.76.

##### Ten-Item Personality Inventory (TIPI)

This inventory is the Polish adaptation by Sorkowska et al. [76,77] and is a widely used, very brief measure of the Big Five personality dimensions (neuroticism, extraversion, conscientiousness, openness to experience, and agreeableness). The questionnaire consists of 10 statements (each trait is assessed by two items). The items are evaluated on a seven-item Likert-type scale from 1 to 7, where the respondents declare if a personality trait applies to them, where 1 = strongly disagree; 2 = moderately disagree; 3 = disagree a little; 4 = neither agree nor disagree; 5 = agree a little; 6 = moderately agree; 7 = strongly agree. The reliability for this scale in the current study was α = 0.67.

In order to collect sociodemographic data, a self-report survey was also used.

#### 4.3.2. Results

Table 6 presents the correlations between the studied variables.

Valence maturity to parenthood was correlated with life values to verify this component of maturity to parenthood validity. Valence maturity to parenthood was positively correlated with tolerance, tradition, and security societal values, and negatively correlated with the face, power—resources, and power—dominance values. The validity of behavioral maturity to parenthood was tested by correlational analyses with resiliency and the willingness to have children. This component of maturity to parenthood was positively correlated with the overall level of resilience and its dimensions: Perseverance and proactive approach, openness to new experiences and sense of humor, personal coping skills and tolerance of negative emotions, tolerance of failures and treating life as a challenge and optimistic attitude to life, and the ability to mobilize oneself in difficult situations. Furthermore, behavioral maturity to parenthood was strongly positively correlated with young adults’ willingness to have children in the future. Cognitive–emotional maturity to parenthood, on the contrary, was positively correlated with active coping and use of support. Moreover, the components of empathy of empathic concern and perspective taking were positively correlated with cognitive–emotional maturity to parenthood. Lastly, the external validity of overall maturity to parenthood was examined by correlational analyses with the relationship satisfaction and personality dimensions. Three of the personality dimensions, namely, extraversion, agreeableness, and conscientiousness, were positively associated with maturity to parenthood and relationship satisfaction.

### 4.4. Discussion

The test–retest correlations supported the strong reliability of the Maturity to Parenthood Scale, confirming the hypothesis 3. Furthermore, by verifying the validity of the MPS, hypotheses H4–H7 were confirmed. 

The validity of valence maturity to parenthood was confirmed by positive correlations with an individual’s life values, such as tolerance, referring to acceptance and understanding of other people who are different from us; tradition, accepting and maintaining the customs, ideas, and traditions of one’s own culture, religion, or family, and respect for tradition; and security societal, a person’s security and stability in society. The VMP subscale also refers to a person’s ethical and moral norms and their coherent and integrated value system. The higher the tolerance, tradition, and security societal, the more likely a person is to present a higher level of valence maturity to parenthood. Furthermore, the validity of VMP was confirmed with negative correlations between VMP and the face, power—resources, and power—dominance values. These values refer to the importance of maintaining and protecting public image and social status in a person’s value system, power over people and resources, and control over people and material and social resources. People who live by these values are reluctant to take on parental roles and are not seen as good parents by society [78,79]. They might find the fulfillment of parental roles difficult and might be at higher risk of psychopathology in parent–child relationships, e.g., by having too much control over a child. In this work, we focused on correlations relevant for checking the validity of the subscales, while the results of this study showed that the personality dimensions values are generally important for maturity to parenthood, not only for the VMP subscale.

In the case of behavioral maturity to parenthood, the results confirmed that this component of maturity to parenthood was positively correlated with the overall level of resilience and its dimensions. Our results also suggest that, besides significant correlations with BMS, resilience was also important for other aspects of maturity to parenthood. Some studies have indicated that resilience can improve parents’ emotional and physical wellbeing [62], which this research confirmed by indicating resilience as an important factor of every aspect of maturity to parenthood—valence, behavioral, and cognitive–emotional. Parenthood is a huge challenge for young adults and a new experience in their life; thus, resiliency is important for coping with different family life challenges related to parenthood [80,81]. Openness to new experiences and sense of humor, tolerance of failures, and treating life as a challenge play important roles in preparing one to becoming a parent. Therefore, more resilient individuals might cope better with the challenges of parenthood in the future. Furthermore, BMP was strongly positively correlated with young adults’ willingness to have children in the future, which also confirmed the convergent validity of the BMS subscale; this is in line with assumptions of the concept that a person who presents a high BMS manages their activities in intimate relationships and in other social relationships, as well as in the economic and work spheres in order to take on parental roles in the future. 

CEMP was positively correlated with two of the components of empathy, namely, empathic concern and perspective taking, but was not related to personal distress. Compassion and caring for others in need and the ability to consider someone else’s point of view are very important for establishing a relationship with a child and for parental responsiveness [8,56,82,83]. Empathy also promotes a positive climate in a family, mutual support between partners, and good communication [5,84,85]. All aspects of maturity are related to the effective regulation of emotions, which is essential for both empathic concern and perspective taking. Maturity to parenthood is thus associated with involvement in social relationships on the basis of care, empathic understanding of others’ needs or problems, and prosocial behaviors or attitudes. Empathic personal distress is associated with problems with emotional regulation and emotional or social disturbances, which might not be connected with the concept of maturity to parenthood. 

The research results also confirmed Hypothesis 7, showing a positive relationship between overall maturity to parenthood (OMP), relationship satisfaction, and personality dimensions. Higher relationship satisfaction is related to greater support, which might facilitate taking on new social roles, including parenting ones [28,53,59]. Only the personality traits of extraversion, agreeableness, and conscientiousness were correlated significantly with OMP, whereas no significant relationships were found with neuroticism and openness to experience. Previous research has indicated that extraversion and agreeableness are associated with greater involvement in parent–child relationships and a higher quality of the above based on support and cooperation [2,18]. There was no negative correlation between OMP and neuroticism, which is commonly described as a predictor of perinatal depression or negative reactions to children [43]. It might be that emotional instability does not significantly affect the vision of future parenthood, instead shaping the transition to parenthood and the fulfillment of parenting roles when real challenges occur, as previous studies have indicated. 

## 5. Conclusions

Maturity to parenthood is an interindividual variable that manifests itself in various spheres of a person’s functioning: the areas of values, behaviors, cognition, and emotions, which may also differ depending on age or sex due to individual developmental and relational factors. This research explored, in two studies, the development, reliability, and validity of the Maturity to Parenthood Scale (MPS), a measure of maturity to parenthood in childless young adults. The MPS is the only questionnaire to date that measures the complex construct of maturity to parenthood, focusing on the extremely important aspect of readiness to take on parental roles in the future. The MPS appears to be a reliable and valid measure comprising the following three subscales: valence, behavioral, and cognitive–emotional maturity to parenthood. The MPS may help to assess maturity to parenthood and to highlight areas where young adults need further support from their relatives or even society. The results also confirmed that both broad- and narrow-band individual differences and contextual factors are important for maturity to parenthood. Taking the perspective of clinical medicine and psychology, the above results may be helpful for judiciary and social welfare (e.g., assessing candidates to be adoptive or foster parents) or medical and psychological care (e.g., helping patients in dealing with decisions to undergo infertility treatment). For example, the MPS is a tool that might be used by health care specialists such as psychologists and therapists in their counselling practice, while working with infertile couples seeking therapeutic support due to stress. Knowledge and reflection on the dimensions of maturity to parenthood might facilitate preparation to parenthood, either biological or adoptive/foster. It might also be useful in designing preventive actions due to a higher risk of developing mood disorders or relationship problems in these couples. Psychoeducation and psychosocial interventions directed at young people should address the issue of maturity to both marriage and parenthood. Raising awareness on the importance of this type of maturity among individuals who include parenting in their plans might support and facilitate their future transitions to parenthood. 

## 6. Limitations and Future Directions

The presented research, despite its valuable results, also has several limitations. In our study, we examined only young Polish adults, which limits the generalizability of the results. Furthermore, women constituted the vast majority of the studied sample; therefore, more men should be examined in future studies. Additionally, the study sample was not representative, mostly consisting of young adults who were well educated and worked, which also limits the generalizability of the results to the population. The Maturity to Parenthood Scale is a self-report questionnaire; thus, people may also be biased when they report on their own experiences. In future studies, it would be interesting to adapt the MPS in other countries and examine if there are intercultural differences in terms of the maturity to parenting in young adults, and to conduct longitudinal studies to investigate if maturity to parenthood assessed with the MPS is related to parental responsiveness when young adults become parents. Conducting research involving young adult couples would also be very interesting and checking the levels of maturity to parenthood in clinical samples and the consequences for the fulfillment of parental roles in the future (longitudinal study).

## Figures and Tables

**Table 1:** Results of the MPS confirmatory factor analysis.

Indices	RMSEA	RMSEA 90% CI	CFI	TLI	*χ*^2^/df
One-factor model (45 items)	0.101	0.099–0.103	0.82	0.82	40,382.081/990
Three-factor model (24 items)	0.079	0.075–0.083	0.94	0.94	19,739.24/276
Four-factor model (45 items)	0.080	0.077–0.082	0.89	0.89	40,382.08/990

**Table 2:** Factor loadings for the three-factor MPS solution.

Factor	Item	Item Loadings
Valence maturity to parenthood	1	0.59 *
	3	0.81 *
	6	0.70 *
	19	0.71 *
	23	0.73 *
	26	0.74 *
	37	0.50 *
Behavioral maturity to parenthood	2	0.81 *
	4	0.60 *
	12	0.60 *
	14	0.54 *
	22	0.55 *
	25	0.73 *
	35	0.74 *
	36	0.72 *
Cognitive–emotional maturity to parenthood	15	0.68 *
	20	0.60 *
	21	0.67 *
	29	0.79 *
	30	0.59 *
	32	0.50 *
	39	0.75 *
	42	0.44 *

* *p* < 0.001.

**Table 3:** Pearson’s correlation matrix of the MPS subscales.

MPS Subscales	1	2	3
1. Valence maturity to parenthood	x		
2. Behavioral maturity to parenthood	0.79 *	x	
3. Cognitive–emotional maturity to parenthood	0.76 *	0.66 *	x

* *p* < 0.001.

**Table 4:** The reliability of the MPS.

Maturity to Parenthood Scale	Cronbach’s α
Valence maturity to parenthood	0.86
Behavioral maturity to parenthood	0.86
Cognitive–emotional maturity to parenthood	0.86
Overall maturity to parenthood	0.94

**Table 5:** Differences in maturity to parenthood depending on the sex of young adults.

	Women		Men		*t*	*p*	Cohen’s *d*
MPS	*M*	*SD*	*M*	*SD*			
Valence	37.76	8.27	35.65	8.76	3.15	0.002	0.25
Behavioral	41.52	10.90	38.34	11.21	3.64	<0.001	0.29
Cognitive–emotional	45.66	7.92	43.92	8.47	2.70	0.007	0.21
Overall	124.94	24.55	117.92	25.73	3.55	<0.001	0.28

**Table 6:** Correlations between studied variable and the MPS.

Variable		Valence Maturity to Parenthood	Behavioral Maturity to Parenthood	Cognitive–Emotional Maturity to Parenthood	Maturity to Parenthood
**PVQ-RR**	Self-directed thought	0.22 **	0.22 **	0.42 **	0.31 **
	Self-directed action	0.27 **	0.22 **	0.45 **	0.33 **
	Stimulation	0.23 **	0.18 **	0.22 **	0.23 **
	Hedonism	0.27 **	0.16	0.37 **	0.28 **
	Achievement	0.23 **	0.19 *	0.40 **	0.29 **
	Power—dominance	−0.25 **	−0.06	−0.03	–0.06
	Power—resources	−0.26 **	−0.05	0.06	–0.02
	Face	−0.25 **	0.13	0.17 *	0.14
	Security Personal	0.25 **	0.23 **	0.29 **	0.28 **
	Security Societal	0.39 **	0.35 **	0.39 **	0.41 **
	Tradition	0.42 **	0.42 **	0.38 **	0.45 **
	Conformity—rules	0.27 **	0.25 **	0.15	0.25 **
	Conformity—interpersonal	0.25 **	0.20 *	0.19 *	0.23 **
	Humility	0.29 **	0.28 **	0.13	0.27 **
	Universalism—nature	0.32 **	0.32 **	0.27 **	0.34 **
	Universalism—concern	0.28 **	0.34 **	0.25 **	0.33 **
	Universalism—tolerance	0.39 **	0.35 **	0.34 **	0.40 **
	Benevolence—care	0.38 **	0.32 **	0.46 **	0.42 **
	Benevolence—dependability	0.29 **	0.24 **	0.45 **	0.35 **
**SPP-25**	Perseverance and proactive approach	0.26 **	0.30 **	0.27 **	0.27 **
	Personal coping skills and tolerance of negative emotions	0.24 **	0.36 **	0.21 **	0.21 **
	Openness to new experiences and sense of humor	0.02	0.36 **	0.12 **	0.06
	Optimistic attitude to life and the ability to mobilize oneself in difficult situations	0.25 **	0.34 **	0.21 **	0.24 **
	Tolerance of failures and treating life as a challenge	0.35 **	0.37 **	0.33 **	0.34 **
	Resiliency	0.33 **	0.39 **	0.33 **	0.32 **
**SWE**	Empathic concern	0.24 **	0.29 **	0.27 **	0.30 **
	Personal distress	−0.07	−0.01	−0.04	–0.04
	Perspective taking	0.15 **	0.22 **	0.22 **	0.22 **
**RELAT**		0.21 **	0.13 **	0.21 **	0.20 **
**TIPI**	Neuroticism	0.14 **	0.03	0.07	0.09
	Extraversion	0.20 **	0.10 *	0.18 **	0.44 **
	Conscientiousness	0.25 **	0.17 **	0.18 **	0.31 **
	Openness to experience	0.11	0.17 *	0.12	0.15
	Agreeableness	0.21 **	0.15 **	0.17 **	0.20 **
**Willingness to have children**		0.46 **	0.45 **	0.39 **	0.46 **

* *p* < 0.05 and ** *p* < 0.01.

## Data Availability

The presented data in this study are available from the corresponding author upon request.

## Appendix A

	**Maturity to Parenthood Scale (MPS)** The following questionnaire consists of various statements about parenting. Please read each sentence and consider to what extent it applies to you. There are no right or wrong answers here. Each answer is correct as long as it is true, i.e., in line with what you think and feel. Please do not omit any line and circle only one of the seven possibilities on each line.	**Strongly Disagree**	**Disagree**	**Somewhat Disagree**	**Neither Agree Nor Disagree**	**Somewhat Agree**	**Agree**	**Strongly Agree**
1	In my life, I would like to experience being a parent.	1	2	3	4	5	6	7
2	I often picture myself playing and caring for my children.	1	2	3	4	5	6	7
3	I know why I would like to become a mother/father.	1	2	3	4	5	6	7
4	I am looking for a full-time job so that I can take maternity and/or parental leave.	1	2	3	4	5	6	7
5	Being a parent is the most beautiful but also the most difficult role in human life.	1	2	3	4	5	6	7
6	I am talking to my partner about parenting.	1	2	3	4	5	6	7
7	I can take care of a baby, such as changing diapers and clothes, washing, and soothing.	1	2	3	4	5	6	7
8	I am open to various scenarios of my/my partner’s pregnancy and childcare (e.g., sick leave during pregnancy vs. work until the end of pregnancy and future mother’s delivery by Caesarean section vs. natural delivery).	1	2	3	4	5	6	7
9	I think I would do well as a mother/father.	1	2	3	4	5	6	7
10	I take into account aging in my plans for parenting.	1	2	3	4	5	6	7
11	I know that the decisions and choices I make will affect my child’s development.	1	2	3	4	5	6	7
12	There are many people among my friends who are parents; therefore, they will surely advise me when I become a parent.	1	2	3	4	5	6	7
13	I have always known that I want to have children.	1	2	3	4	5	6	7
14	When buying a flat on a loan, I will take into account the enlargement of my family.	1	2	3	4	5	6	7
15	I believe that I will be able to reconcile parenthood with the other roles that I fulfill in life, and it will give me complete happiness.	1	2	3	4	5	6	7
16	At the end of my life, I want to be proud that I have raised a good person/people.	1	2	3	4	5	6	7
17	I know that when a baby is born, it can be harder than I imagine, and the characteristics of a baby may surprise me.	1	2	3	4	5	6	7
18	The role models I have in my parents will allow me to raise a happy child.	1	2	3	4	5	6	7
19	The thought of parenting responsibilities is pleasing to me.	1	2	3	4	5	6	7
20	I am interested in what equipment is needed for childcare to be functional and safe.	1	2	3	4	5	6	7
21	I will choose my professional future so that I can combine parenting with work.	1	2	3	4	5	6	7
22	I believe that neither money nor career are important in life, and the most important thing is family.	1	2	3	4	5	6	7
23	I would like to show my children the world and teach them all I can.	1	2	3	4	5	6	7
24	In raising my child, I will try to avoid the mistakes that my parents made.	1	2	3	4	5	6	7
